# Satellite communications in health emergencies: no longer a luxury

**DOI:** 10.5365/wpsar.2025.16.1.1217

**Published:** 2025-03-27

**Authors:** Pierre-Yves Beauchemin, Eystein Grusd, Erin E Noste, Anthony Cook, Jan-Erik Larsen, Aristides Poblete Alonzo, Misheel Enkhdalai, Sean T Casey

**Affiliations:** a World Health Organization Regional Office for the Western Pacific, Manila, Philippines.; b Department of Emergency Medicine, University of California San Diego, San Diego, California, United States of America.; c School of Population Health, Faculty of Medicine and Health, University of New South Wales, Sydney, New South Wales, Australia.

In health emergencies, communication is a lifeline. Rapid coordination and information-sharing are critical for needs assessments, resource allocation and effective emergency response. In recent years, satellite communications have evolved from being prohibitively expensive to becoming an essential and affordable tool for emergency responders. Recent disasters, such as the 7.3 magnitude earthquake and resulting temporary communications blackout in Vanuatu on  17 December 2024, and a similar event following the 2022 volcanic eruption and tsunami in Tonga, underscore the importance of reliable satellite communications during emergencies. (*1*-*5*) In both instances, terrestrial cellular and data networks were down for the first several days after the event, and only those with access to satellite communications devices could contact counterparts and partners outside of the affected areas.

When disasters strike, traditional networks often collapse, leaving satellite communications as the only reliable option. (*6*) Tools such as satellite telephones, satellite messaging, geolocation devices and portable satellite internet devices generally remain operational, even in the most remote areas or when conventional networks are unavailable. These enable critical tasks, such as relaying information, coordinating medical referrals and logistics, addressing urgent needs and enhancing coordination through real-time data-sharing. These benefits were demonstrated by satellite communications devices in the hours and days following the December 2024 earthquake in Vanuatu, when the Ministry of Health and the National Disaster Management Office, the World Health Organization (WHO) and other United Nations agencies, as well as other partners, leveraged satellite technology to call for and coordinate international assistance.

Satellite communications, once costly and complex, are now both affordable and user-friendly. Subscription costs are similar to those of cellular phones and terrestrial mobile and data service providers, and many suppliers offer flexible plans that can be easily changed when needed following initial activation. WHO has procured satellite communications devices for some countries to strengthen emergency response communications readiness. However, in several cases, upon follow-up, these devices have remained inactive months after delivery, limiting their operational impact when emergencies arise. There are a few reasons for this. While basic costs may be modest – approximately US$ 150 per year for standby (limited use) satellite messaging – government or partner budgeting delays, administrative bottlenecks and lack of routine maintenance (e.g. software updates) sometimes prevent these devices from being deployment-ready. Moving forward, emergency responders should integrate satellite communications into field deployment kits and standard operating procedures from the outset. Devices must be activated, tested regularly and maintained, with clear budgeting for subscriptions and updates. Governments and partners must proactively finance and facilitate ongoing maintenance and address operational barriers to ensure that satellite devices are fully functional and ready to deploy when they’re needed the most. Routine monitoring or drills are needed to confirm the readiness of this critical resource.

Satellite communications are no longer a luxury: they are a critical component of disaster preparedness and emergency responses, helping to save lives and ensure efficient coordination. However, their use remains inconsistent across countries and partners. Prioritizing procurement, subscriptions for maintenance and training for personnel can help to ensure that satellite communications support more effective health emergency responses in the future.
